# Matrix-assisted laser desorption-ionization-time-of-flight mass spectrometry as a reliable proteomic method for characterization of *Escherichia coli* and *Salmonella* isolates

**DOI:** 10.14202/vetworld.2017.1083-1093

**Published:** 2017-09-19

**Authors:** Waleed S. Shell, Mahmoud Lotfy Sayed, Fatma Mohamed Gad Allah, Fatma Elzahraa Mohamed Gamal, Afaf Ahmed Khedr, A. A. Samy, Abdel Hakam M. Ali

**Affiliations:** 1 Central Laboratory for Evaluation of Veterinary Biologics Abbasaia, Agriculture Research Center, Cairo, Egypt; 2 Department of Microbiology and Immunology, National Research Center, Cairo, Egypt

**Keywords:** ABI, Bruker Daltonics, colibacillosis, *Escherichia coli*, matrix-assisted laser desorption ionization time-of-flight mass spectrometry, *Salmonella*, *Salmonella* pullorum

## Abstract

**Aim:**

Identification of pathogenic clinical bacterial isolates is mainly dependent on phenotypic and genotypic characteristics of the microorganisms. These conventional methods are costive, time-consuming, and need special skills and training. An alternative, mass spectral (proteomics) analysis method for identification of clinical bacterial isolates has been recognized as a rapid, reliable, and economical method for identification. This study was aimed to evaluate and compare the performance, sensitivity and reliability of traditional bacteriology, phenotypic methods and matrix-assisted laser desorption-ionization-time-of-flight mass spectrometry (MALDI-TOF MS) in the identification of clinical *Escherichia coli* and *Salmonella* isolates recovered from chickens.

**Materials and Methods:**

A total of 110 samples (cloacal, liver, spleen, and/or gall bladder) were collected from apparently healthy and diseased chickens showing clinical signs as white chalky diarrhea, pasty vent, and decrease egg production as well as freshly dead chickens which showing postmortem lesions as enlarged liver with congestion and enlarged gall bladder from different poultry farms.

**Results:**

Depending on colonial characteristics and morphological characteristics, *E. coli* and *Salmonella* isolates were recovered and detected in only 42 and 35 samples, respectively. Biochemical identification using API 20E identification system revealed that the suspected *E. coli* isolates were 33 out of 42 of colonial and morphological identified *E. coli* isolates where *Salmonella* isolates were represented by 26 out of 35 of colonial and morphological identified *Salmonella* isolates. Serological identification of isolates revealed that the most predominant *E. coli* serotypes were O1 and O78 while the most predominant *Salmonella* serotype of *Salmonella* was *Salmonella* Pullorum. All *E. coli* and *Salmonella* isolates were examined using MALDI-TOF MS. In agreement with traditional identification, MADI-TOF MS identified all clinical bacterial samples with valid scores as *E. coli* and *Salmonella* isolates except two *E. coli* isolates recovered from apparently healthy and diseased birds, respectively, with recovery rate of 93.9% and 2 *Salmonella* isolates recovered from apparently healthy and dead birds, respectively, with recovery rate of 92.3%.

**Conclusion:**

Our study demonstrated that Bruker MALDI-TOF MS Biotyper is a reliable rapid and economic tool for the identification of Gram-negative bacteria especially *E. coli* and *Salmonella* which could be used as an alternative diagnostic tool for routine identification and differentiation of clinical isolates in the bacteriological laboratory. MALDI-TOF MS need more validation and verification and more study on the performance of direct colony and extraction methods to detect the most sensitive one and also need using more samples to detect sensitivity, reliability, and performance of this type of bacterial identification.

## Introduction

*Escherichia coli* infection in poultry is one of the principal causes of mortality and morbidity in chickens and turkeys resulting in great economic losses to poultry industry due to, retardation of growth, decreased feed conversion rate, decreased egg production, decreased fertility, reduced hatchability, downgraded carcasses and condemnation of whole affected carcasses or organs after slaughter and finally the high cost of wide range of antibacterial agents used to control *E. coli* infection in many poultry farms [1]. Colibacillosis in chickens refers to local and systematic (extraintestinal) infections caused mainly by avian pathogenic *E. coli* [2], which are commonly belong to certain O groups, particularly O1, O2, O8, O15, O18, O35, O78, O88, O109, and O115 [3]. *E. coli* infection in poultry is responsible for a variety of disease conditions such as colisepticemia, air sac disease, serositis (peritonitis, pericarditis, and perihepatitis), omphalitis, panophthalmitis, synovitis, salpingitis, coligranuloma, swollen head syndrome, cellulitis, yolk sac infection, and enteritis [4].

One of most common economically important bacterial disease in poultry industry is Salmonellosis particularly fowl typhoid and pullorum disease [5]. Avian *Salmonella* infection is caused by different *Salmonella* species [6]. More than 2500 *Salmonella* serotypes have been mentioned under the species but only about 10% of these serotypes have been isolated from poultry [7]. Among this, *Salmonella* Pullorum (SP) species (*S. enterica* subsp. enterica serovar pullorum) which causing pullorum disease and *Salmonella* Enterica serovar Gallinarum is main causative agent of fowl typhoid.

The bacteriological method for detecting clinical bacterial isolates as *Salmonella* and *E. coli* involves culturing the organism in different specific and selective media and identifying isolates using traditional and conventional bacteriological methods is time-consuming. Therefore a rapid, sensitive, specific, reliable, and cost effective method for identification of pathogens in clinical samples is required. As an alternative to various other identification methods, mass spectral (proteomics) analysis for identification of clinical bacterial isolates has been recognized. Matrix-assisted laser desorption-ionization-time-of-flight mass spectrometry (MALDI-TOF MS) can be used as a sensitive, reliable and rapid procedures for identification of various clinical bacterial isolates [8], such as Gram-positive bacteria [9], mycobacteria [10], *Brucella* [11], *Enterobacteriaceae* [8], yeast [12], mold [13], and non-fermenting bacteria [14].

The aim of this study is to evaluate and compare the performance, reliability, and sensitivity of classical bacteriological and phenotypic methods in comparison to MALDI-TOF MS in identification of *E. coli* and *Salmonella* recovered from chickens.

## Materials and Methods

### Ethical approval

All samples were collected as per standard sample collection procedure without giving any stress or harm to the animals. Such type of study do not require any specific ethical approval.

### Sampling

A total of 110 samples collected from different poultry farms including apparently healthy (31 cloacal swabs), and diseased (49 cloacal swabs) chickens which showing clinical signs as white chalky diarrhea, pasty vent, and decrease egg production and also from freshly dead chickens (30 liver, spleen, and gallbladder samples) which showing postmortem lesions as enlarged liver with congestion and enlarged gallbladder. The samples were transferred immediately to sterile buffered peptone water, then wrapped with ice, kept in box and transferred directly to the lab [15].

### Isolation of E. coli and Salmonella isolates

Isolation of *E. coli* and *Salmonella* was carried out on three successive stages which are pre-enrichment in non-selective liquid broth [15], enrichment in selective liquid media [16] and plating onto solid selective agar media as MacConkey agar, SS agar and eosin methylene blue (EMB) agar media [17].

### Identification of E. coli and Salmonella isolates

#### Colonial and microscopical examination E. coli and Salmonella isolates

The suspected colonies were examined for their colonial morphology [15] on nutrient agar, EMB agar, MacConkey agar, xylose lysine decarboxylase agar (XLD), and *Salmonella*-Shigella agar (S-S). Microscopical examination was performed according to Merchant and Packer [18]. Isolates were preserved for further examination by growing and spreading of the microorganism by stabbing in semisolid agar [19]. Isolates were tested for motility [20].

#### Biochemical identification of E. coli and Salmonella isolates

Biochemical identification of isolates was done using pure cultures of each of the suspected isolates using API 20E plate system (Biomerieux –France cat# 20-100).

#### Serological identification of E. coli and Salmonella isolates

Serological identification of the isolates was conducted according to Kauffmann [21]. Smooth colonies of *E. coli* isolates that were preliminary identified biochemically as *E. coli* were subjected to serological identification according to Sojka [22], Edward and Ewing [23] against the polyvalent 1, 2, 3, and 4 antisera using the agglutination test. These polyvalent antisera are:

•Polyvalent (1): O1, O26, O86, O111, O119, O127, O128
•Polyvalent (2): O2, O11, O87, O127, O142
•Polyvalent (3): O6, O27, O78, O148, O159, O168
•Polyvalent (4): O44, O55, O125, O126, O146, O166.

The positive agglutinating isolates with the polyvalent antisera was retested with corresponding specific monovalent antisera. These monovalent antisera are:

O1, O26, O86, O111, O119, O127, O128. O2, O11, O87, O127, O142, O6, O27, O78, O148, O159, O168, O44, O55, O125, O126, O146, O166.

Smooth culture of biochemically identified *Salmonella* isolates was further tested using polyvalent and monovalent *Salmonella* antisera O and H factor using slide agglutination [21,23].

### MALDI-TOF MS (extraction method) [24,25]

One to 2 pure colonies of *E. coli* or *Salmonella* were suspended in 300 ul of molecular grade water (Sigma-Aldrich, St. Louis, MO) and vortexed. Then, 900 ul of absolute ethanol was added, vortexed, and centrifuged at 20,800 ×*g* for 3 min. The supernatant was decanted, and the pellet was dried at room temperature then, 50 ul of 70% formic acid and 50 ul of acetonitrile were added and mixed by pipetting, followed by centrifugation at 20,800 ×*g* for 2 min. 2 ul of supernatant was applied into the 24 spot plate and left to dry at room temperature followed by the addition of 2 ul of MALDI matrix (a saturated solution of -cyano-4-hydroxycinnamic acid in 50% acetonitrile and 2.5% trifluoroacetic acid). For each plate, a bacterial test standard (Bruker Daltonics) was included to calibrate the instrument and validate the run. Spectra were analyzed using MALDI Biotyper automation control and the Bruker Biotyper 2.0 software and library (version 2.0, 3,740 entries; Bruker Daltonics). Identification score criteria were performed as recommended by Bruker Daltonics which evaluated as follow:

•A score of 2.000 indicated species level identification
•A score of 1.700-1.999 indicated identification to the genus level
•A score of 1.700 was interpreted as no identification.

With respect of direct isolation of causative agents as a gold standard test, API 20A and MALDI-TOF MS sensitivity, relative sensitivity and specificity in identification of causative agents were calculated using (https://www.medcalc.org/calc/diagnostic_test.php) as shown in Table-1.

**Table-1:** Calculation of sensitivity and specificity with respect of gold standard test (https://www.medcalc.org/calc/diagnostic_test.php).

Results	Gold standard test (cft)		Total

	Positive	Negative	
Test under evaluation			
Positive	A	B	A+b
Negative	C	D	C+d
Total	A+c	B+d	n (264)

Relative sensitivity=A/A+C, specificity=D/D+B, true positive (positive predictive value)=A/A+B, false positive (B)=B/A+B, true negative (negative predictive value)=D/D+C, false negative (C)=C/D+C

## Results and Discussion

### Isolation and identification of E. coli and Salmonella isolates

In birds, *E. coli* infections cause many clinical manifestations; the most common is being airsacculitis, pericarditis, septicemia, and death [26]. Colibacillosis due to virulent *E. coli* in chickens is characterized by a respiratory disease which is frequently followed by a generalized infection [27]. *Salmonellae* are widespread in human and animals worldwide. In industrialized countries, non-typhoid *Salmonellae* is an important cause of bacterial gastroenteritis. Zoonotic *Salmonella* Enterica serovars are among the most important agents of food-borne infections throughout the world. Poultry is one of the major sources of *Salmonella*-contaminated food products that cause human Salmonellosis [28].

In this study, a total of 110 samples were collected from apparently healthy (31 cloacal), diseased (49 cloacal), and freshly dead (30 liver and hearts) chickens from different poultry farms and examined microbiologically.

### Colonial characteristics and morphological characteristics of the E. coli and Salmonella isolates

Depending on colonial characteristics and morphological characteristics, *E. coli* was detected in only 42 clinical specimens. These isolates were 11 out of 31 isolates recovered from apparently healthy chickens, 17 out of 49 isolates recovered from diseased chickens, and 14 out of 30 isolates recovered from freshly dead chickens. Suspected *E. coli* isolates when cultured on different media were showed rounded, non-pigmented colonies on nutrient agar medium, while on MacConkey agar medium showed rounded, non-mucoid pink colonies (lactose fermenter). At the same time, the same isolates on SS agar appeared as rounded, non-mucoid pink colonies and on EMB agar showed a distinctive yellow-green metallic sheen. These isolates were Gram-negative, motile, non-sporulated, and medium-sized bacilli (Table-2). Whereas, 35 suspected isolates were behaved as *Salmonella* spp. and were aerobic and facultatively anaerobic, have a wide temperature range and like all enterobacteria grow readily on all ordinary media. On MacConkey agar, *Salmonella* colonies were 2-4 mm in diameter and pale since lactose was not fermented after 18-24 h incubation at 37°C while on SS agar, *Salmonella* appeared transparent with black centers. In the same time on XLD agar, *Salmonella* appeared pink with black pigment indicating H_2_S production. These isolates were Gram-negative non-spore-forming medium size straight rods and usually motile (Table-2). All above-mentioned results agree with Antunes *et al*. [29] and Ozbey and Ertas [15].

**Table-2:** *E. coli* and *Salmonella* isolates recovered from different samples.

Source	Number of samples	Number of suspected *E. coli* isolates	Number of suspected *Salmonella* isolates
Apparently healthy	31	11	9
Diseased	49	17	17
Freshly dead	30	14	9
Total	110	42	35

*E. coli=Escherichia coli*

### Biochemical identification of E. coli and Salmonella isolates

Depending on the results of API 20E identification system, the suspected *E. coli* isolates were 8 out of 11 apparently healthy samples, 14 out of 17 diseased samples, and 11 out of 14 freshly dead samples representing recovery rates of 73%, 82%, and 79%, respectively (Table-3), where 6 suspected *Salmonella* isolates were recovered from 9 of apparently healthy samples, 13 isolates out of 17 diseased samples, and 7 isolates out of 9 freshly dead samples representing recovery rates of 67%, 76%, and 78%, respectively (Table-4).

**Table-3:** Biochemical characteristics of the suspected *E. coli* isolates using API20E system.

Type of samples	Number of samples	API 20E results																					Number of recovered isolates		Recovery rate (%)

		ONPG	ADH	LDC	ODC	CIT	H2S	URE	TDA	IND	VP	GEL	GLU	MAN	INO	SOR	RHA	SAC	MEL	AMY	ARA	OX			
Apparently healthy	11	+	-	+	+	-	-	-	-	+	-	-	+	+	-	+	+	+	+	-	+	-	4	8	73
		+	-	+	-	-	-	-	-	+	-		+	+	-	+	+	-	+	-	+	-	2		
		+	+	+	+	-	-	-	-	+	-	-	+	+	-	+	+	+	+	-	+	-	2		
Diseased	17	+	-	+	+	-	-	-	-	+	-	-	+	+	-	+	+	+	+	-	+	-	5	14	82
		+	-	+	-	-	-	-	-	+	-	-	+	+	-	+	+	-	+	-	+	-	3		
		+	+	+	+	-	-	-	-	+	-	-	+	+	-	+	+	+	+	-	+	-	4		
		+	-	-	-	-	-	-	-	+	-	-	+	+	-	+	+	+	+	-	+	-	2		
Freshly dead	14	+	-	+	+	-	-	-	-	+	-	-	+	+	-	+	+	+	+	-	+	-	3	11	79
		+	-	+	-	-	-	-	-	+	-	-	+	+	-	+	+	-	+	-	+	-	2		
		+	+	+	+	-	-	-	-	+	-	-	+	+	-	+	+	+	+	-	+	-	3		
		+	-	-	-	-	-	-	-	+	-	-	+	+	-	+	+	+	+	-	+	-	3		
Total	42																							33	

*E. coli=Escherichia coli*, ONPG=Ortho nitro phenyl-βD-galactopyranosidase, ADH=Arginine dihydrolase, LDC=Lysine decarboxylase, ODC=Ornithine decarboxylase, CIT=Citrate utilization, H2S=Hydrogen sulfide, URE=Urease, TDA=Tryptophan deaminase, IND=Indole, VP=Voges Proskauer, GEL=Gelatinase, GLU=Glucose (fermentation/oxidation), MAN=Mannitol (fermentation/oxidation), INO=Inositol (fermentation/oxidation), SOR=Sorbitol (fermentation/oxidation), RHA=Rhamnose (fermentation/oxidation), SAC=Saccharose (fermentation/oxidation), MEL=Melibiose (fermentation/oxidation), AMY=Amygdalin (fermentation/oxidation), ARA=Arabinose (fermentation/oxidation), OX=Oxidase

**Table-4:** Biochemical characteristics of the suspected *Salmonella* isolates using API20E system.

Type of samples	Number of samples	API results																					Recovered number of isolates		Recovery rate (%)

		ONPG	ADH	LDC	ODC	CIT	H2S	URE	TDA	IND	VP	GEL	GLU	MAN	INO	SOR	RHA	SAC	MEL	AMY	ARA	OX			
Apparently healthy	9	-	-	+	+	+	+	-	-	-	-	-	+	+	+	+	+	-	+	-	+	-	2	6	67
		-	+	+	+	+	+	-	-	-	-	-	+	+	-	+	+	-	+	-	+	-	4		
Diseased	17	-	-	+	+	+	+	-	-	-	-	-	+	+	+	+	+	-	+	-	+	-	4	13	76
		-	+	+	+	+	+	-	-	-	-	-	+	+	-	+	+	-	+	-	+	-	3		
		-	+	+	+	+	+	-	-	-	-	-	+	+	+	+	+	-	+	-	+	-	6		
Freshly dead	9	-	-	+	+	+	+	-	-	-	-	-	+	+	+	+	+	-	+	-	+	-	2	7	78
		-	+	+	+	+	+	-	-	-	-	-	+	+	-	+	+	-	+	-	+	-	2		
		-	+	+	+	+	+	-	-	-	-	-	+	+	+	+	+	-	+	-	+	-	3		
Total	35																							26	

ONPG=Ortho nitro phenyl-βD-galactopyranosidase, ADH=Arginine dihydrolase, LDC=Lysine decarboxylase, ODC=Ornithine decarboxylase, CIT=Citrate utilization, H2S=Hydrogen sulfide, URE=Urease, TDA=Tryptophane deaminase, IND=Indole, VP=Vagous Proskauer, GEL=Gelatinase, GLU=Glucose (fermentation/oxidation), MAN=Mannitol (fermentation/oxidation), INO=Inositol (fermentation/oxidation), SOR=Sorbitol (fermentation/oxidation), RHA=Rhamnose (fermentation/oxidation), SAC=Saccharose (fermentation/oxidation), MEL=Melibiose (fermentation/oxidation), AMY=Amygdalin (fermentation/oxidation), ARA=Arabinose (fermentation/oxidation), OX=Oxidase

### Serological identification of E. coli and Salmonella isolates

Tables-5 and 6 summarized serotyping of *E. coli* and *Salmonella* isolates using polyvalent and monovalent antisera. Most of *E. coli* strains were belonging to serotype O1 and O78 were the most predominant serotype of *Salmonella* strains was SP.

**Table-5:** Serogrouping of the suspected *E. coli* isolates.

Source	Apparently healthy	Diseased	Freshly dead	Total	Recovery rates (%)
Number of isolates	8	14	11	33	
Polyvalent antisera					
1	4	2	3	9	
2	0	2	1	3	
3	4	7	5	16	
4	0	2	3	5	
Monovalent antisera					
O1	4	3	2	9	27.3
O2	0	2	1	3	9.1
O6	2	3	2	7	21.2
O78	2	4	3	9	27.3
O126	0	2	3	5	15.1

*E. coli=Escherichia coli*

**Table-6:** Serotyping of the suspected *Salmonella* isolates.

Source	Apparently healthy	Diseased	Freshly dead	Total	Recovery rates (%)
Number of isolates	6	13	7	26	
SP	2	4	2	8	30.8
SM	1	1	0	2	7.7
SE	3	2	2	7	26.9
SG	0	2	1	3	11.5
ST	0	4	2	6	23.1

SP=*Salmonella* Pullorum, SM=*Salmonella* Montevideo, SE=*Salmonella* Enteritidis, SG=*Salmonella* Gallinarum, ST=*Salmonella* Typhimurium

It was surprising that the identified *E. coli* samples of the same source showed variations in their biochemical reactions, this may be due to difference in serotypes of these identified samples. Kwon *et al*. [30] identified *E. coli* isolates by screening biochemical traits using API 20E identification system. Regarding serodifferentiation, chicken may harbor many different serotypes in their gastrointestinal tract, in this study, only a restricted number of serotypes O1, O2, O6, O78, and O126 have been recovered. These results were confirmed by Salama *et al*. [31] who recovered 5 different *E. coli* serotypes identified as O1, O2, O6, O78, and O126. Pathogenic *E. coli* isolates for poultry commonly belong to certain serogroups, particularly the serogroups O78, O1, and O2, and sometimes O15 [32,33]. The relation between biochemical and serological identification of *E. coli* confirmed that the variation of reactions in between the same source of samples was related to the difference in serotypes and also revealed the similarity between serotypes O1 and O2 in their biochemical reactions [34]. Similar serotypes (O1, O2, and O78) were obtained by Chart *et al*. [33], McPeake *et al*. [35]. In addition, Peighambari *et al*. [36], Lafont *et al*. [37], Dho-Moulin *et al*. [38], and Gross [39] recorded that the most common serogroups of *E. coli* from avian diseases were O78, O2, and O1 which were associated with septicemic *E. coli* infection in poultry. Furthermore, Cloud *et al*. [40] and Orajaka and Mohan [41] recorded a high incidence of serovars O1, O2, and O78 in case of colibacillosis. Furthermore, Hossain *et al*. [42] recorded that out of 110 bird samples, 66 samples were found to be positive for *E. coli* meanwhile Robab and Azadeh [43], isolated 50 *E. coli* strains from bile and liver of poultry. All the isolated and identified bacteria possess the morphological, biochemical and serological characteristics of *E. coli* and the O1 and O78 serotypes are the most predominated. On the other hand, Raji *et al*. [44] isolated *E. coli* from hatcheries and the most common serovares were O8, O9 and O78 among poultry cases. Kilic *et al*. [45] isolated *E. coli* from 110 samples collected from colibacillosis suspicious hens at different poultry farms in a recovery rate of 48%. Serogroup O1 is known pathogen in poultry and usually isolated from birds with colibacillosis [46]. Rosenberger *et al*. [47] reported that O2 serovars of avian origin are among virulent avian *E. coli* in colibacillosis. The isolation of O6 serotype which usually cause septicemic diarrhea in newborn and enteritis in domestic animals is evidence that the water sources of the farms were probably contaminated with sewage and/or the farms laborers did not observe sanitary measures [48].

For *Salmonella* isolation and identification, Moustafa [49] reported that the predilection seats for isolation of *Salmonella* were the genital organs, spleen, gallbladder, and liver while intestinal contents or feces were not reliable for *Salmonella* isolation. Furthermore, Bygrave and Gallagher [50] isolated *Salmonella* Enteritidis (SE) from pooled samples of liver, lungs, testes, cecum, and intestine. Zahraei *et al.*, [17] isolated 30 *Salmonella* species from intestine and liver of chicken in poultry farms using SS agar and xylose-lysine deoxycholate agar after enriching on selenite-f broth. Further, serological identification of the suspected colonies was applied using the polyvalent and monovalent antisera. The results revealed that five serotypes of *Salmonella* were isolated represented by SP, *Salmonella* Typhimurium (ST), SE, *Salmonella* Gallinarum, and *Salmonella* Montevideo (SM). These results were confirmed by Chaiba *et al*. [51] who used poultry samples and identified four different *Salmonella* serotypes which are ST, *Salmonella* Newport, SM, and *Salmonella Heidelberg* using polyvalent O and H antisera.

### MADI-TOF MS identification of E. coli and Salmonella isolates

Using MADI-TOF MS, all microscopical, morphological, biochemical and serological identified *E. coli*, and *Salmonella* isolates were tested. MADI-TOF MS identified all clinical bacterial samples as *E. coli* and *Salmonella* except two *E. coli* isolates recovered from apparently healthy and diseased birds, respectively, with recovery rate of 93.9% and 2 *Salmonella* isolates recovered from apparently healthy and dead birds, respectively, with recovery rate of 92.3%. 3 out of these 4 isolates were had un-valid score (red color) where the 4^th^ sample which isolated from apparently healthy bird and bacteriologically identified as *E. coli* were identified with a valid score as Pseudomonas fragi using MALDI-TOF MS (Table-7). For more accuracy of the results, the samples being processed and spotted in duplicates and consequences the reproducibility of MALDI-TOF MS apparatus was evaluated and found to be consistent for all bacterial clinical samples [52,53]. Preparatory extraction is superior to direct colony method for the bacterial identification by MALDI-TOF MS using the Bruker system also using the extraction method increased identification to the species level [28,54].

**Table-7:** Identification of *E. coli* and *Salmonella* field isolates using MALDI-TOF.

Analyte ID	Organism (best matched)	Matched pattern	Score value	NCBI identifier
EA1	*E. coli*	*E. coli* DH5alpha BRL+*E. cloacae* MB_8779_05 THL	2.362	562
EA2	*E. coli*	*E. coli* DH5alpha BRL+*E. kobei* DSM 13645T DSM	2.493	562
EA3	*E. coli*	*E. coli* W3350 MMG+*E. fergusonii* DSM 13698T HAM	2.1	562
EA4	*E. coli*	*E. coli* W3350 MMG+*K. cowanii* DSM 18146T DSM	2.448	562
EA5	*P. fragi*	*P. fragi* DSM 3456T HAM+*P. jessenii* CIP 105274T HAM	2.325	296
EA6	*E. coli*	*E. coli* ATCC 25922 THL+*C. koseri* DSM 4570 DSM	2.57	562
EA7	*E. coli*	*E. coli* W3350 MMG+*C. farmeri* CCUG 29877 CCUG	2.36	562
EA8	*E. coli*	*E. coli* ATCC 25922 THL+*C. koseri* DSM 4570 DSM	2.66	562
EDS1	*E. coli*	*E. coli* DH5alpha BRL+*E. hormaechei* ssp hormaechei DSM 12409T DSM	2.573	562
EDS2	*E. coli*	*E. coli* W3350 MMG+*E. fergusonii* DSM 13698T HAM	2.494	562
EDS3	*E. coli*	*E. coli* DH5alpha BRL+*E. cloacae* MB_8779_05 THL	2.095	562
EDS4	Not reliable identification	*E. coli* ATCC 25922 CHB	1.585	562
EDS5	*E. coli*	*E. coli* W3350 MMG+*K. cowanii* DSM 18146T DSM	2.345	562
EDS6	*E. coli*	*E. coli* ATCC 25922 THL+*C. koseri* DSM 4570 DSM	2.675	562
EDS7	*E. coli*	*E. coli* DH5alpha BRL+*E. cloacae* MB_8779_05 THL	2.278	562
EDS8	*E. coli*	*E. coli* DH5alpha BRL+*E. kobei* DSM 13645T DSM	2.354	562
EDS9	*E. coli*	*E. coli* W3350 MMG+*K. cowanii* DSM 18146T DSM	2.476	562
EDS10	*E. coli*	*E. coli* ATCC 25922 THL+*C. koseri* DSM 4570 DSM	2.133	562
EDS11	*E. coli*	*E. coli* DH5alpha BRL+*E. cloacae* MB_8779_05 THL	2.464	562
EDS12	*E. coli*	*E. coli* DH5alpha BRL+*E. cloacae* MB_8779_05 THL	2.565	562
EDS13	*E. coli*	*E. coli* DH5alpha BRL+*E. kobei* DSM 13645T DSM	2.467	562
EDS14	*E. coli*	*E. coli* ATCC 25922 THL+*C. koseri* DSM 4570 DSM	2.423	562
EDE1	*E. coli*	*E. coli* DH5alpha BRL+*E. kobei* DSM 13645T DSM	2.575	562
EDE2	*E. coli*	*E. coli* W3350 MMG+*K. cowanii* DSM 18146T DSM	2.257	562
EDE3	*E. coli*	*E. coli* ATCC 25922 THL+*C. koseri* DSM 4570 DSM	2.165	562
EDE4	*E. coli*	*E. coli* ATCC 25922 THL+*C. koseri* DSM 4570 DSM	2.298	562
EDE5	*E. coli*	*E. coli* DH5alpha BRL+*E. kobei* DSM 13645T DSM	2.376	562
EDE6	*E. coli*	*E. coli* ATCC 25922 THL+C. koseri DSM 4570 DSM	2.256	562
EDE7	*E. coli*	*E. coli* W3350 MMG+*E. fergusonii* DSM 13698T HAM	2.237	562
EDE8	*E. coli*	*E. coli* W3350 MMG+*E. fergusonii* DSM 13698T HAM	2.237	562
EDE9	*E. coli*	*E. coli* DH5alpha BRL+*E. kobei* DSM 13645T DSM	2.312	562
EDE10	*E. coli*	*E. coli* ATCC 25922 THL+*C. koseri* DSM 4570 DSM	2.296	562
EDE11	*E. coli*	*E. coli* ATCC 25922 THL+*C. koseri* DSM 4570 DSM	2.276	562
SA1	Not reliable identification	*Salmonella* sp. (choleraesuis) 08 LAL	1.328	591
SA2	*Salmonella*	*Salmonella* sp. (enterica st Dublin) Sa05_188 VAB	2.134	98,360
SA3	*Salmonella*	*Salmonella* sp. (enterica st Enterica) DSM 17058T HAM+*E. coli* MB11464_1 CHB	2.328	59,201
SA4	*Salmonella*	*Salmonella* sp. (enterica st Hadar) Sa05_506 VAB+*E. coli* W3350 MMG	2.425	149,385
SA5	*Salmonella*	*Salmonella* sp. (enterica st Enterica) DSM 17058T HAM+*E. hormaechei* ssp hormaechei DSM 12409T DSM	2.294	59,201
SA6	*Salmonella*	*Salmonella* sp. (choleraesuis) 08 LAL+*E. coli* ATCC 25922 CHB	2.118	591
SDS1	*Salmonella*	*Salmonella* sp. (enterica st Gallinarum) FLR+*C. sakazakii* DSM 4485T DSM	2.051	594
SDS2	*Salmonella*	*Salmonella* sp. (enterica st Enterica) DSM 17058T HAM+*K. pneumoniae* ssp pneumoniae 9295_1 CHB	2.366	59,201
SDS3	*Salmonella*	*Salmonella* sp. (enterica st Gallinarum) FLR+*K. pneumoniae* ssp pneumoniae 9295_1 CHB	2.361	594
SDS4	*Salmonella*	*Salmonella* sp. (enterica st Anatum) 11 LAL+*C. koseri* 9553_1 CHB	2.386	58,712
SDS5	*Salmonella*	*Salmonella* sp. (enterica st Hadar) Sa05_506 VAB+*E. coli* ATCC 25922 THL	2.346	149,385
SDS6	*Salmonella*	*Salmonella* sp. (enterica st Enterica) DSM 17058T HAM+*K. cowanii* DSM 18146T DSM	2.413	59,201
SDS7	*Salmonella*	*Salmonella* sp. (enterica st Enterica) DSM 17058T HAM+*K. cowanii* DSM 18146T DSM	2.333	59,201
SDS8	*Salmonella*	*Salmonella* sp. (enterica st Hadar) Sa05_506 VAB+*E. coli* ATCC 25922 THL	2.268	149,385
SDS9	*Salmonella*	*Salmonella* sp. (enterica st Anatum) 11 LAL+*C. koseri* 9553_1 CHB	2.236	58,712
SDS10	*Salmonella*	*Salmonella* sp. (enterica st Hadar) Sa05_506 VAB+*E. coli* W3350 MMG	2.578	149,385
SDS11	*Salmonella*	*Salmonella* sp. (enterica st Gallinarum) FLR+*C. sakazakii* DSM 4485T DSM	2.378	594
SDS12	*Salmonella*	*Salmonella* sp. (enterica st Enterica) DSM 17058T HAM+*E. hormaechei* ssp hormaechei DSM 12409T DSM	2.319	59201
SDS13	*Salmonella*	*Salmonella* sp. (enterica st Enterica) DSM 17058T HAM+*K. pneumoniae* ssp pneumoniae 9295_1 CHB	2.372	59201
SDE1	*Salmonella*	*Salmonella* sp. (enterica st Enterica) DSM 17058T HAM+*E. hormaechei* ssp hormaechei DSM 12409T DSM	2.333	59201
SDE2	*Salmonella*	*Salmonella* sp. (enterica st Dublin) Sa05_188 VAB	2.224	98,360
SDE3	Not reliable identification	*Salmonella* sp. (choleraesuis) 08 LAL	1.211	591
SDE4	*Salmonella*	*Salmonella* sp, (enterica st Anatum) 11 LAL+*C. koseri* 9553_1 CHB	2.328	58,712
SDE5	*Salmonella*	*Salmonella* sp. (enterica st Enterica) DSM 17058T HAM+*E. coli* MB11464_1 CHB	2.239	59,201
SDE6	*Salmonella*	*Salmonella* sp. (enterica st Dublin) Sa05_188 VAB	2.334	98,360
SDE7	*Salmonella*	*Salmonella* sp. (enterica st Hadar) Sa05_506 VAB+*E. coli* W3350 MMG	2.106	149,385

EA=*E. coli* isolate recovered from apparently healthy birds, EDS=*E. coli* isolate recovered from diseased birds, EDE=*E. coli* isolate recovered from dead birds, SA=*Salmonella* isolate recovered from apparently healthy birds, SDS=*Salmonella* isolate recovered from diseased birds, SDE=Salmonella isolate recovered from dead birds, *E. cloacae=Enterobacter cloacae*, *E. kobei=Enterobacter kobei*, *E. fergusonii=Escherichia fergusonii*, *K. cowanii=Kosakonia cowanii*, *P. fragi=Pseudomonas fragi*, *P. jessenii=Pseudomonas jessenii*, *C. koseri=Citrobacter koseri*, *C. farmeri=Citrobacter farmeri*, *E. hormaechei=Enterobacter hormaechei*, *E. cloacae=Enterobacter cloacae*, *C. sakazakii=Cronobacter sakazakii*, *K. pneumoniae=Klebsiella pneumoniae*, *E. coli=Escherichia coli*

**Figure-1:**
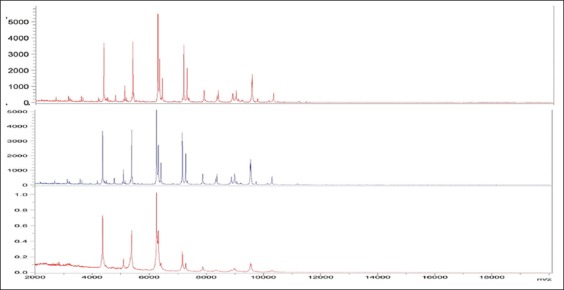
Overview of the matrix-assisted laser desorption-ionization-time-of-flight mass spectra of 3 *Escherichia coli* field isolates.

**Figure-2:**
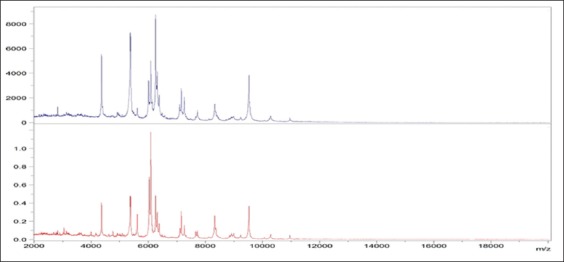
Overview of the matrix-assisted laser desorption-ionization-time-of-flight mass spectra of 3 *Salmonella* Gallinarum field isolates.

Valid identification scores as explained by Bruker Daltonik MALDI Biotyper is 2.0 or more were enough for a reliable identification to the species level (green color) which mean highly probable species identification (2.300-3) or secure genus identification, probable species identification (2-2.299) where score 1.700-1999 and 0.000-1.699 means probable genus identification (yellow color) and not reliable identification (red color), respectively [55,56]. By examination of *E. coli* and *Salmonella* isolates and strains revealed from apparently healthy, diseased and dead chickens by MALDI-TOF MS, 10-20 prominent ion peaks were identified in the mass spectra. Range of these prominent ion peaks were from the 3000 and 10,500 m/z, with the highest-intensity peaks being in the range of 4375-9625 m/z with *E. coli* isolates while in the case of *Salmonella* isolates, range of these spectra peaks were from the 3000 and 11,000 m/z, with the highest-intensity spectra peaks being in the range of 4350-9500 m/z. On this basis, the score values achieved by MALDI-TOF MS correctly identified all *E. coli* and *Salmonella* isolates at the species level (score ≥2.0). Inspection of mass spectra reveals strain-specific peaks at 4375, 5375, 6650, 7190, and 9625 m/z for all *E. coli* isolates which agree with Christner *et al*. [57] and also reveals strain-specific peaks at 4350, 5300, 5600, 6090, 6200, 6300, 7200, 7750, 8500, and 9500 m/z for all *Salmonella* isolates which agree to large extent with Dieckmann and Malorny [58] and Leuschner *et al*. [59], respectively (Figures-1 and 2).

In our study, MALDI-TOF MS gave a valid score for genus and species identification of 93.94% when used in identification of previously identified *E. coli* culture using ABI system and conventional methods this agrees with Ge *et al*. [60], Jesumirhewe *et al*. [61], and Naiara *et al*. [62] which achieved species identification of *E. coli* isolates using MALDI-TOF MS of 94.7%, 80%, and 83%, respectively, when compared with traditional methods of identification. All this studies not identified *E. coli* to sub species level. On the other hand, Huixia *et al*. [63] was developed a rapid method to identify *E. coli* at subspecies level (identifying flagellar (H) antigen) using a MALDI-TOFMS platform with high sensitivity and specificity which could identify 100% of reference strains containing H types (53 strains) and could detect 75 out of 85 clinical isolates representing matched results obtained from traditional serotyping.

Furthermore, pure colonies previously identified as *Salmonella* isolates using ABI system and traditional methods gave valid score of 91.66% using MALDI-TOF MS assay. This results agrees with Ulrich *et al*. [64] which reported that no positive sample was missed by this novel approach which allowed detection of pure *Salmonella* culture after just 1 day of incubation and also agrees with Rebecca *et al*. [65] which found that MALDI-TOF MS could identified 98% of *Salmonella* clinical samples that previously identified by traditional methods. Public Health England [66], Clark *et al*. [67] and Kuhns *et al*. [68] reported that MALDI-TOF MS has been used to help in both detection and species-level identification of *Salmonella* and also has been utilized in discriminating *Salmonella* Enterica serovar Typhi from other *Salmonella* serovars (subspecies level).

Results revealed that there is no satisfactory differences were observed in and sensitivity (positive cases/total number of suspected cases × 100) of 20A and MALDI-TOF MS when compared with direct isolation of causative agents as sensitivity in case of *E. coli* were 78.57% and 73.8%, respectively, wherein case of *Salmonella* 74.29% and 68.57%, respectively, where sensitivity of MALDI-TOF MS in compression of API 20A was 93.93% and 92.3% in case of *E. coli* and *Salmonella* isolates, respectively. With respect of direct isolation of causative agents as a gold standard test, relative sensitivity, and specificity were 100% and 88.31% with API 20A and 100% and 86.08% with MALDI-TOF, respectively, in case of *E. coli* isolates where in case of *Salmonella* isolates, relative sensitivity, and specificity of API 20A were 100% and 89.29% and of MALDI-TOF MS were 100% and 87.21%, respectively. With respect of API 20A, relative sensitivity, and specificity of MALDI-TOF MS were 100% and 81.82%, respectively, in the case of *E. coli* and *Salmonella* isolates.

MALDI-TOF MS showed significant promise in *E. coli* and *Salmonella* identification on genus and species levels and can be also used as a tool for sub species and serovar typing, but it will require additional studies and modifications to existing protocols and commercial and the extended database. The identification using MALDI-TOF MS method could analyze pure positive culture rapidly (may be within minutes especially when direct cultural identification methods used rather than ethanol: Formic acid extraction method) and also reliable manner. However, identification by traditional methods needs more facilities, media, chemicals, experiences, and time and this in contrast with the non-requirement of high technical expertise, the simple extraction procedure and low running cost identification using MALDI-TOF MS which provide more advantages over other methods for identification. However, the applications have to be carried out with cautions because the accuracy decreases using of too much of chemicals and materials and the samples have to be spotted with the matrix solution with care to avoid the presence of the liquid smear between spots, which increase possibility of cross-contamination [69,70]. The sample size used for this study is low as it is a preliminary study to use this technique in diagnostic laboratories in Egypt, but anyhow, more samples are needed in future studies to detect sensitivity, reliability, and performance of this type of bacterial identification.

## Conclusion

This study demonstrated that Bruker MALDI-TOF MS Biotyper is a reliable fast and economic tool for the identification of Gram-negative bacteria, especially *E. coli* and *Salmonella* which could be used as alternative regular diagnostic tool for routine identification and differentiation of clinical isolates in the bacteriological laboratory to provide more precise identification on clinical specimens. MALDI-TOF MS need more validation and verification and more study on the performance of direct colony and extraction methods to detect the most sensitive one and also need using more samples to detect sensitivity, reliability, and performance of this type of bacterial identification.

## Authors’ Contributions

All authors designed and planned this research work. Isolation of causative agents from field and preparation of samples for MALDI-TOF analysis were done by WSS, MLS and AAS. Biochemical and serological identification were done by FMGA, FEMG and AAK. All authors contributed equally in preparation and revision of the manuscript and collection of scientific papers related to the subject of this research. All authors read and approved the final manuscript.
